# Artificial Intelligence and Health Equity: Opportunities and Obstacles

**DOI:** 10.1016/j.jacadv.2024.101045

**Published:** 2024-07-03

**Authors:** Rahul C. Deo

**Affiliations:** a Division of Cardiovascular Medicine for Brigham and Women’s, Department of Medicine for Harvard Medical School, Brigham and Women’s Hospital and Harvard Medical School, Boston, Massachusetts, USA; b Atman Health, Inc, Needham Heights, Massachusetts, USA

**Keywords:** artificial intelligence, diagnosis, health equity, treatment

Artificial intelligence (AI) has the potential to democratize medicine, thereby helping overcome longstanding inequities in health care. In this viewpoint, I will examine how such a change may arise while emphasizing regulatory and commercial barriers to sustainable adoption. My focus will be on the United States, partly due to personal familiarity, including having spent several years building a health technology startup, and partly because device regulation and economic models of value in health care vary dramatically across countries.

To narrow the scope further, it is worth defining the democratization of care. In this case, I will limit the goal to consistent, high-quality cardiovascular care, regardless of a patient’s place of residence or socioeconomic status. Although cardiovascular care encompasses many facets, including procedural interventions, our focus will be excellence in diagnosis and nonprocedural treatments.

What are the main obstacles to achieving equity? Many studies have cited specialist access as a significant barrier.^1^ Beyond access, there is variability in care—reflected in poor guideline attainment^2^ and, in some cases, low diagnosis rates for less common diseases.^3^ Finally, there are evidence gaps for specific subpopulations due to low clinical trial enrollment or simply many clinical decisions that have never been systematically evaluated.^4^ There are other vital concerns, including perceived or actual bias in decision-making (human or AI model-based),^5^ lack of trust in the health care system, and unaddressed social determinants of health—but I will not attempt to address these in this current viewpoint.

I will move successively through personnel specialization levels, starting with how AI can help cardiologists, then seeking to shift more of their work to noncardiologist providers, and then exploring contributions by nonlicensed personnel and autonomous agents. Lastly, I will explore evidence generation within a software-driven clinical workflow. For each proposed solution, I will discuss the expectations of regulatory agencies and whether the economic value is compelling enough to imagine sustainable uptake in the market.

I start with perhaps the most straightforward example—AI-based over-reading electrocardiograms and echocardiograms. The motivation for this application, similar to computer-aided detection for mammograms, which has been around for nearly 2 decades, is the idea that certain features of clinical importance may be missed and that machines may be well-suited to be a tireless and consistent second pair of eyes. One common target for AI models is (treatable) rare diseases, which, in cardiology, include such conditions as cardiac amyloidosis, hypertrophic, and pulmonary arterial hypertension.^6^ It has been hypothesized that such diagnoses are more likely to be missed in community settings than specialty referral centers, primarily because of a much lower prevalence. Convolutional neural network models are well-suited to increase detection for such diseases, although post-test probability challenges exist. From a health equity perspective, it is evident that a patient with one of these treatable rare diseases should be diagnosed expeditiously no matter where they might live so that they may then be directed to places with expertise to treat them.

How does the United States Food and Drug Administration (FDA) view such an application? The risk seems low to many, given that this is merely assisting (and not replacing) a specialist who already possesses the skill to carry out this task. And the model's output is usually one piece of the decision-making process. However, these models typically have been classified as class II devices by the FDA. The critical distinction is that software that cannot provide a fully transparent and human-reproducible step-by-step basis for a decision (from input to output) is viewed as a device.^7^ The FDA offers an extensive list of examples of devices that cover all models taking continuous signals as inputs (continuous glucose monitoring, electrocardiogram, continuous heart rate), all image processing, and all neural network-based models. Attempting to bring a device to market necessitates the device manufacturer going through an arduous submission process to seek FDA clearance. Academic publications typically are insufficient as evidence, and one is expected to perform an adequately powered multi-institutional study (retrospective or prospective) to validate model performance across diverse populations while implementing quality management systems for software development. The intended clinical use must be unambiguous (who, what, when, why), and additional studies are typically needed to have intended users go through example use cases to alleviate safety concerns that may arise. Unsurprisingly, the cost of such studies can be hundreds of thousands or millions of dollars.

Given the sizable investment needed to bring just one such model to the market, is there a sound economic case for accelerating the identification of patients with rare diseases? Unfortunately, for most centers or practice groups (and startups), finding a financial justification is often not straightforward. Without delving into the full complexity of payer contracts, we can focus on the extremes of fee-for-service activity, where one is paid for what one does, and value-based care, where one is rewarded by delivering high-quality care at a low price. In the fee-for-service realm, identifying a small number of patients with a rare disease is not likely to have much financial impact. In general, population-level patient identification programs are not standard. Still, when they occur, they are judged by downstream revenue, which in cardiology is typically procedural and depends on absolute patient numbers, which is hard to justify for rare diseases. One exception may be if deploying the AI model itself can be reimbursed. Given that a single center may screen hundreds of thousands of studies each year with a single model with minimal marginal cost, this could be a sizeable source of revenue, though there are few examples of this to date. In the value-based care realm, additional treatment costs of newly identified patients, which can be sizable for rare disease therapies, must be offset by savings in resource utilization that arise from a timely diagnosis. So, even though there is a substantial health equity case for this AI application and good reason to believe it can be successful, the economic and regulatory factors may slow the adoption of these approaches. Implicit in this discussion is the sobering fact that excellent medicine, whether diagnosis or treatment, does not always have a business case behind it.

Our first example focused on achieving equity in the quality of care (in particular, diagnostic interpretation) but did not address the fact that in many parts of the country, having access to a cardiologist is the primary problem. Can AI address this by shifting cardiologists' work toward other licensed health care professionals? Today, mid-level practitioners are already extending cardiologists in some aspects of patient care. And in other countries where specialists are even more scarce, we see general internists and primary care physicians taking on more nonprocedural aspects of cardiac care. What would be needed to fully replace the nonprocedural aspects of cardiology?

It is helpful to think about treatment and diagnosis separately. In the case of treatment, there are often well-described guidelines for management decisions. Such guidelines are lengthy and frequently change with new evidence, and it can be taxing for a nonspecialist to keep up with them.^8^ However, it is feasible for a machine to help in this space, surfacing recommendations that are up to date with the latest evidence. Revisiting the regulatory angle, if the basis for such decisions is transparent and can be reviewed, reproduced, and sourced, such software can currently avoid regulation as a device by the FDA—and could be classified as clinical decision support.^7^ Would there be an economic argument for doing so? Generally, yes. Delivering comparable care at a lower cost is usually rewarded under all possible payer models.

However, the path is not that simple. One challenge with AI addressing physician shortage by augmenting the expertise of a nonspecialist is that it is difficult to separate just one activity (in this case, the medication treatment aspect) from all the other tasks that a specialist does. So, our AI-assisted mid-level practitioner may not find much demand for the ability to carry out guideline-driven treatment at a specialist level without also dealing with the influx of new diagnostic problems to work-up and other miscellaneous responsibilities (eg, preoperative clearance) that may arise.

This problem, where AI can only help with a portion of a job that is not easily separable from other activities, evokes the proposed use of AI-assisted echocardiogram data acquisition.^9^ The vision for such a solution is that in situations where sonographers are scarce, nonsonographers can acquire most routine echocardiographic views. The challenge is that when acquiring data, sonographers also recognize when there is an atypical finding and acquire a series of more specialized views to ensure the abnormality is well-characterized. It is far more challenging to train an AI solution to replicate this serial process, which is much more hypothesis driven and interactive. So, we may have a solution that does 75 to 80% of what is needed at a reduced cost but cannot carry out the remainder and may not even be able to tell when that remainder is required.

The examples I have given are deliberately circumscribed and represent examples of solutions that are already going through regulatory submissions or have entered the commercial market and are wrestling with making a case for value. However, many have far greater ambitions for AI in medicine, including some version of an AI doctor who can exhibit excellent diagnostic acumen and decision-making. In such a world, the problem of provider shortages would be markedly alleviated. Today, we already have autonomous conversational agents that can take a patient's report of symptoms, work through a differential diagnosis, and propose further diagnostic testing or a treatment plan.^10^ Although such an agent depends on external inputs, it could be coupled with a person (or robot) who could help acquire physical exam findings, which a machine could interpret.

However, given the existing regulatory framework, these agents would undoubtedly be classified as devices. And the scope of any FDA submission for regulatory approval would be vast. How could we convince a regulatory body that all potential eventualities will be handled adequately, given that the possible range of input-output scenarios is infinite? In many ways I see this as being like obtaining approval for deploying self-driving cars, which required years of driving data encompassing millions of miles. The up-front investment for such model validation would be enormous (for autonomous vehicles, this has been estimated in the tens of billions of dollars), and one can imagine requiring hundreds of thousands of patient encounters collected under study protocols to understand the full range of safety and efficacy. And just as with self-driving cars, although the overall safety profile will eventually be excellent, there remains the possibility of alarming, incomprehensible errors that will not be acceptable to the public.

The scenarios above all started with the presumption that medical evidence is sufficient, and to achieve health equity, we merely need to focus on delivering it consistently. However, this assumption is suspect. In many cases, the low participation rate of minorities in clinical trials leaves some uncertainty over the applicability of guidelines to these subpopulations. And in other cases, the basis for guidelines may be weak.^4^ To some, machine learning implies machines learning what people know, but a broader vision for machine learning is for machines to learn what is unknown. The processes above that describe the systematization of model training and deployment, continuous validation, and iterative improvement hint at a learning health care system that can address high-value evidence gaps, including those for the most vulnerable populations who may not be well represented in current guidelines.

Despite highlighting obstacles to implementing AI models in practice, I am optimistic that it can contribute to overcoming health inequities. However, within the realities of the current regulatory and economic framework, the path to sustainable adoption will require ingenuity and strategic partnerships among all stakeholders.

## Funding support and author disclosures

Dr Deo is a cofounder of Atman Health, a company focused on software-driven acceleration of guideline-directed medical therapy in cardiovascular, kidney, and metabolic disease.
